# Cancer Cell Fusion: Mechanisms Slowly Unravel

**DOI:** 10.3390/ijms17091587

**Published:** 2016-09-21

**Authors:** Felicite K. Noubissi, Brenda M. Ogle

**Affiliations:** 1 Department of Biology, Jackson State University, Jackson, MS 39217, USA; felicite.noubissi_kamdem@jsums.edu; 2 Department of Biomedical Engineering, University of Minnesota-Twin Cities, Minneapolis, MN 55455, USA; 3 Stem Cell Institute, University of Minnesota-Twin Cities, Minneapolis, MN 55455, USA; 4 Masonic Cancer Center, University of Minnesota-Twin Cities, Minneapolis, MN 55455, USA; 5 Lillehei Heart Institute, University of Minnesota-Twin Cities, Minneapolis, MN 55455, USA; 6 Institute for Engineering and Medicine, University of Minnesota-Twin Cities, Minneapolis, MN 55455, USA

**Keywords:** cell fusion, genomic instability, phosphatidyl serine receptor, metastasis, genetic diversity

## Abstract

Although molecular mechanisms and signaling pathways driving invasion and metastasis have been studied for many years, the origin of the population of metastatic cells within the primary tumor is still not well understood. About a century ago, Aichel proposed that cancer cell fusion was a mechanism of cancer metastasis. This hypothesis gained some support over the years, and recently became the focus of many studies that revealed increasing evidence pointing to the possibility that cancer cell fusion probably gives rise to the metastatic phenotype by generating widespread genetic and epigenetic diversity, leading to the emergence of critical populations needed to evolve resistance to the treatment and development of metastasis. In this review, we will discuss the clinical relevance of cancer cell fusion, describe emerging mechanisms of cancer cell fusion, address why inhibiting cancer cell fusion could represent a critical line of attack to limit drug resistance and to prevent metastasis, and suggest one new modality for doing so.

## 1. Introduction

Approximately 90% of cancer-related deaths are caused by the local invasion and distant metastasis of tumor cells. Metastasis is arguably the most poorly understood aspect in cancer. To successfully relocate in the body, a tumor cell must acquire transient properties that enable dissemination, followed by the reestablishment of the original primary phenotype at a distant site. Exactly how this is accomplished is still unclear, and reliable treatments are therefore lacking. One hypothesis suggests that a variety of genetic and epigenetic changes lead to the development of breast cancer. These changes involve somatic gene mutations, copy number aberrations, exon sequencing changes, alterations in miRNA and protein expression levels, and changes in methylation [1,2,3,4]. Hence, the unstable cancer genome combined with host selective pressures generates metastatic cells in the otherwise non-metastatic primary tumor [5]. This view continues to provide some framework for envisioning tumor progression. However, it is difficult to imagine how this might occur through successive, stepwise mutations, as the generation of a metastatic phenotype would require the activation and silencing of large numbers of genes in the primary tumor cell. Moreover, a recent report compared the entire genome of a primary tumor cell with a corresponding metastatic tumor cell, and found only two de novo mutations in the metastatic tumor with neither mutation essential to the metastatic process [6]. A second widely accepted paradigm for cancer progression is that epithelial cells undergo a mesenchymal transition, during which they lose apical-basal polarity and intercellular adhesions, and express mesenchymal genes such as *N*-cadherin and vimentin. Then, single mesenchymal cells escape from the epithelial tumor mass and enter the lymphatic system or bloodstream, through which they disseminate. At ectopic sites in the body, the tumor cells extravasate, revert to an epithelial phenotype, and colonize surrounding tissue to form metastases [7,8]. However, epithelial-to-mesenchymal transition (EMT) is not essential for tumor invasion, as epithelial cells can collectively invade [9,10]. Furthermore, circulating tumor cells isolated from cancer patients show the expression of markers for both mesenchymal and epithelial cells [11,12]. A third more recent hypothesis suggests that the tumor bulk contains a heterogeneous tumor cell population that is derived from a subset of cells that show the characteristics of stem cells, termed tumor-initiating cells or cancer stem cells (CSCs) [13,14]. They are capable of dividing asymmetrically to produce one stem cell, which enables self-renewal, and one progenitor cell, which allows the production of phenotypically-diverse cancer cells that constitute tumors. The CSCs might result from the deregulation of normal stem cell self-renewal and differentiation pathways [14,15,16], or may develop from EMTs [17,18]. This current idea has yet to be universally adopted, as the origin of CSCs is still controversial. A fourth possibility (which is the topic of this review) stipulates that the fusion of tumor cells with cells of hematopoietic lineage or stromal lineage gives rise to hybrid cells capable of dissemination and new tumor growth. The possibility that cell fusion gives rise to the metastatic phenotype was first put forward nearly a century ago by Aichel [19], and later on by Mekler [20] and Goldenberg [21]. Since then, the hybrid theory has been proposed as an explanation for tumor metastasis [22,23,24]. In this review, we will present various studies pointing to the contribution of cancer cell fusion to metastasis, the possible role of cancer cell fusion in chemoresistance, and some potential mechanisms governing cancer cell fusion.

## 2. Cell Fusion and Metastasis

Several in vitro and in vivo studies have shown that metastatic cells result from the fusion of primary tumor cells and cells of hematopoietic lineage [24,25,26,27] or other cell types of the tumor microenvironment [28,29,30,31]. These fusion events were shown to occur spontaneously in many cases. For instance, spontaneous fusion was observed in vitro between normal breast epithelium and breast cancer cells [28,29,30,31], among breast tumor cells themselves [32], between breast cancer epithelium and endothelial cells [33], between breast cancer epithelium and stroma cells [22,34,35], and between lung cancer cells and stroma cells [36]. Further analysis of hybrids resulting from these spontaneous fusion events showed that they harbored metastatic properties. For example, hybrids formed between normal breast epithelium (M13SV1-EGFP-Neo) and breast cancer cells (HS578T-Hyg) showed increased locomotory activity compared to the normal parental line. This fusion-enhanced migration was associated with altered CCL21/CCR7 signaling, which was previously linked to the metastatic spreading of breast cancer to lymph nodes [28]. We found in our studies that breast tumor cells could spontaneously fuse with mesenchymal stem cells (MSCs) to form hybrids presenting increased invasion and migratory capacity [34,37]. 

The increased metastatic potential of hybrids was also observed in vivo when breast cancer cell variants (MDA-MB-231) with tropism for either lung or bone injected in nude mice gave rise to hybrids capable of metastases to both organs [32]. Moreover, fusion was detected when freshly-mixed lung cancer cells and MSCs were xenografted by subcutaneous injection into nonobese diabetic severe combined immunodeficient (NOD/SCID) mice [36]. The hybrids formed acquired epithelial-to-mesenchymal (EMT) properties and increased motility and invasiveness. They also displayed stem cell markers and were found to contribute to highly-malignant subpopulations enriched for lung cancer-initiating cells [36]. Additionally, cells of a melanoma clone (wild type for tyrosinase, C/C) implanted into BALB/c nu/nu mice (homozygous mutation for albino tyrosinase, c/c) developed massive pulmonary metastases a few weeks later. Analysis of chromosomes of cells from the metastatic tumors showed that most clones had acquired the c allele (the same as that of the BALB/c recipient), while maintaining the C allele. Thus, lung metastases were comprised primarily of host-tumor hybrids; interestingly, these hybrids expressed the same traits of enhanced motility and MSH/BMX responsiveness as in vitro-derived melanoma-macrophage hybrids [38]. The motility-associated integrin subunits α2, α3, α5, α6, αv, β1, and β3—which are involved with the migration of leucocytes and cancer cells—were significantly upregulated in metastatic macrophage-melanoma hybrids compared with parental melanoma cells. They also produced high levels of β1,6-branched oligosaccharides—predictors of poor survival in patients with melanoma or carcinomas of the breast, lung, and colon [39,40]. A more recent study also indicated that fusion between cancer cells (ovarian and lung) and hematopoietic cells of the myeloid lineage gave rise to hybrids expressing significantly higher levels of the promigratory marker C-X-C chemokine receptor type 4 (CXCR4) that was conferred by the parental myeloid cells [23].

Collectively, the increase in motility-associated integrin subunits and CXCR4 levels in hybrids might equip them with superior migratory potential and help their dissemination to various secondary organs, and therefore explain how fusion could provide a means by which adherent cancer cells acquire new qualities necessary to form metastases (i.e., enhanced motility and matrix degradation) under conditions conducive to hematopoietic survival, and later resume tumor-like activities (i.e., rapid proliferation) under conditions conducive to epithelial survival. Another potent example of cancer cell fusion-driven metastasis is the study by Li et al. [41] that showed that human hepatocellular carcinoma cells with low metastatic potential exhibited significantly increased metastatic potential following fusion with MSCs, as proven by the gross examination of tumors. The parental hepatocellular carcinoma cells induced well-differentiated noninvasive tumors, whereas fused cells induced poorly-differentiated and invasive tumors when implanted in mouse liver.

Clinical studies have also confirmed the presence of cell fusion in tumors. This was first demonstrated in patients who had received hematopoietic stem cell transplantation and later on developed tumors showing evidence of donor genes in their cells [42,43]. Other discoveries in cancer patients of circulating tumor cells expressing both carcinoma and leucocyte cell markers also points to fusion events between bone marrow-derived cells and tumor cells [25,44,45]. More recently, a study found macrophage-melanoma hybrids in the peripheral blood of patients with cutaneous melanomas. The study further demonstrated that those hybrids, when transplanted subcutaneously in nude mice, produced metastatic lesions at distant sites [46]. Those hybrids in patients’ peripheral blood might have been on their way to distant sites to develop metastases. Another recent report used short tandem repeat length-polymorphism and forensic genetic techniques to show that a metastatic melanoma lesion in a patient arose from the fusion between a tumor cell and a bone marrow-derived cell that the patient received as a transplant [47]. Xu et al. [36] also proposed that, after fusion between cancer cells and bone marrow-derived cells, the hybrids undergo EMT that facilitates their migration and invasion and also the acquisition of stem cell-like properties that enhance tumorigenicity and the ability to metastasize. As time goes by, however, those hybrids reacquire epithelia-like morphology by a process termed mesenchymal-epithelial transition, or MET. Thus, cell fusion could encompass or account for both the first transition from cell–cell or cell–matrix bound to unattached, and the second transition from blood transit to proliferation at the point of metastasis. However, although cancer cell fusion is seen in cancer patients and some metastases, and though it represents an attractive mechanism to explain the process of metastasis, it has yet to be clearly demonstrated in vivo that cancer cell fusion in the primary tumor is at the origin of metastasis.

## 3. Cancer Cell Fusion and Tumor Heterogeneity

Cancer is a clinically and genomically heterogeneous disease (reviewed in [48]). This heterogeneity is observed between tumors as well as within individual tumors [49]. This diversity in the populations of tumor cells is thought to be responsible for the emergence of subpopulations resistant to treatment and the development of metastasis. Although the origin of heterogeneity in a given patient is likely multifaceted, fusion between tumor cells and cells of the tumor microenvironment might represent a key mechanism generating the critical population diversity needed to evolve resistance to therapy and metastasis. Studies have suggested that the fusion of cells in general (and fusion between cancer cells and cells of the tumor microenvironment especially) is a means to generate widespread genetic and epigenetic diversity [19,27,38,40,50,51,52]. Diversity created in this way could rapidly enhance the formation, propagation, and metastasis of tumor cells, or quickly alter drug sensitivity. Close examination of the heterogeneity generated by the heterotypic formation of stromal cell–breast cancer cell hybrids indicated that hybrids exhibited mixed gene expression profiles and could undergo DNA ploidy reduction and morphologic switching from mesenchymal-like to breast carcinoma-like. In addition, analysis of coding single-nucleotide polymorphisms by RNA sequencing revealed genetic contributions from both fusion partners to primary tumors and metastasis [22].

A recent study proposed a potent explanation of cell fusion-driven heterogeneity and metastasis mechanism. Zhou et al. [53], used normal intestinal crypt epithelial cells from rats to demonstrate that cell fusion generates populations of cells in which about 1 of 200 could form tumors. In addition, fusion engenders aneuploidy, DNA damage, phenotypic heterogeneity, transformation, and the capacity to form tumors, and these properties were established immediately or within a few cell divisions after the fusion event. They found that tumors formed after the implantation of fusion-derived clones obtained from the same parental line exhibited distinct rates of growth and histology. Some of the clones generated rapidly-growing high-grade undifferentiated tumors, whereas other clones formed slowly-growing moderately differentiated tumors exhibiting a high degree of invasiveness. Overall, these studies support the premise that cell fusion events induce increases in genetically diversified cell populations.

## 4. Cancer Cell Fusion and Chemoresistance

The diversity of tumor cell populations could account for the drug susceptibility found to be altered in hybrids. Hybrids formed between parental breast cancer cells (MCF-7) with and without resistance to doxyrubicin were heterogeneous in nature; some exhibiting resistance, and others not [54]. Similarly, hybrids derived from breast epithelial cells (M13SV1-EGFP-Neo) and breast cancer cells (MDA-MB-435-Hyg) showed altered sensitivity to the phosphoinositide 3-kinase (PI3K) inhibitor Ly294002 as a consequence of differential RAF-AKT (Rapidly Accelerated Fibrosarcoma-Akt) crosstalk among hybrids [31]. Moreover, studies by Wang [55] showed that the fusion of stem cells and liver cancer cells generated hybrids which were highly tumorigenic and chemoresistant compared with the parental hepatocellular carcinoma cells. Co-cultivation of mouse bone marrow-derived cells and murine 67NR mammary carcinoma cells resulted in the origin of cells exhibiting markedly increased expression levels of the ABC multidrug resistance transporters Abcb1a and Abcb1b, associated with an enhanced resistance towards chemotherapeutic drugs [35]. Thus, the fusion theory offers a compelling explanation for the tumor heterogeneity-driven emergence of cells resistant to chemotherapeutics.

Early detection of the sub-population of treatment resistant and/or metastasis-prone cancer cells and the characterization of residual metastatic cancers would significantly improve cancer management, as it would direct the course of treatment appropriate for the patient. Precision medicine and personalized therapy based on the identification of the molecular drivers of cancer by genome sequencing has been proposed as a strategy to overcome the effects of tumor heterogeneity on metastasis [48,49,56]. This approach has been proven beneficial in various cancers (chronic myeloid leukemia, breast, melanoma, colorectal, and others; reviewed in [57]), however targeted cancer cells can quickly develop resistance to the drug. In addition, not all types of cancer have personalized treatment, and are still under exploration as cutting-edge tools and technologies are developed to identify critical targets implicated in their progression. Another limitation of precision medicine is that not all identified targets that modulate cancer progression are druggable. Moreover, there are a large number of genomic and epigenomic aberrations that have been discovered in cancer. It would be extremely expensive to try to develop targeted therapy for each of those aberrations. Targeted therapy attacks tumor heterogeneity after it is established. However, tackling tumor heterogeneity before inception could represent a more advantageous approach. This could be achieved by preventing cancer cell fusion. Molecular mechanics of hybrid formation could therefore present prime targets, and might include (1) members of pathways that facilitate close apposition of cell membranes of fusing partners; (2) specific cell surface receptors involved in cell–cell contact-induced fusion; (3) members of pathways that govern the integration of parental fusion partner genes into hybrid genomes; (4) epigenetic modifications enabling transcript expression of hybrid genomes; and even (5) immune modulation to disable or enhance macrophage fusion [58].

## 5. Potential Molecular Mechanisms and Signaling Pathways Driving Cancer Cell Fusion

The mechanisms governing cancer cell fusion are still underexplored, and this review represents a call for increased study in this area. Here we consider elements that have been unraveled and consider their possible interplay to facilitate tumor cell fusion. Our studies showed that fusion between MSCs and breast tumor cells was significantly increased in hypoxic condition, and was regulated by a mechanism involving apoptosis. We found that the inhibition of apoptosis reduced cell fusion, whereas the addition of apoptotic cells to co-cultures could significantly enhance fusion [34]. A previous work also identified apoptotic cells as a new type of cue that promotes fusion of myoblasts by inducing signaling via the phosphatidylserine receptor BAI1 (brain specific angiogenesis inhibitor 1) pathway [59]. Myoblasts and macrophages have been shown to use some of the same molecular components in fusion, and in these studies, activation of BAI1 triggers ELMO/Dock180/Rac1-associated pathways [60].

**Figure 1:**
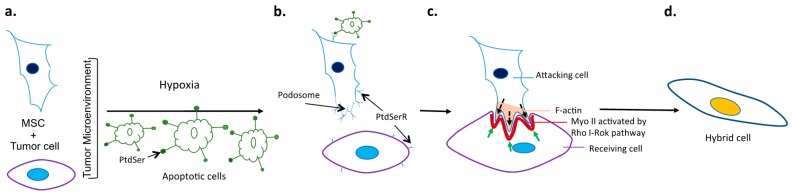
Unraveling and targeting mechanisms of cancer cell fusion. (**a**) Tumor cells and mesenchymal stem cells (MSCs) are capable of spontaneous fusion, which is augmented with hypoxia; (**b**) Fusion in hypoxic conditions can be facilitated by the engagement of the exposed phosphatidyl serine (PtdSer) of apoptotic cells with PtdSer receptors (PtdSerR) on tumor cells or MSCs. Engagement of this type facilitates podosome formation that ultimately leads to robust activation of the F actin of the attacking fusion partner and MyoII of the receiving cell; (**c**) Green arrows indicate resisting forces from the actomyosin network, and black arrows indicate pushing forces from invasive protrusions of the attacking cell; (**d**) Hybrids formed in this way represent an accelerated evolution of sorts, sometimes giving rise to cells with enhanced metastatic potential or the ability to resist drug treatment. Inhibiting the engagement of apoptotic cells via PtdSer represents one potential therapeutic approach to the prevention of tumor cell fusion.

ELMO/Dock180/Rac proteins are a conserved signaling module for the promotion of the internalization of apoptotic cell corpses. ELMO and Dock180 function together as a guanine nucleotide exchange factor (GEF) for the small GTPase Rac, and thereby regulate the phagocyte actin cytoskeleton during engulfment [61]. BAI1 was identified as a receptor upstream of ELMO, and as a receptor that can bind PtdSer on apoptotic cells. BAI1 forms a trimeric complex with ELMO and Dock180, and functional studies suggest that BAI1 cooperates with ELMO/Dock180/Rac to promote the maximal engulfment of apoptotic cells. Decreased BAI1 expression or interference with BAI1 function was shown to inhibit the engulfment of apoptotic targets ex vivo and in vivo. Thus, BAI1 is a PtdSer recognition receptor that can directly recruit a Rac-GEF complex to mediate the uptake of apoptotic cells [62]. ELMO and Dock180 are overexpressed in breast cancer cell lines [63]. Activation of the ELMO-Dock signaling pathway has also been shown to be involved in breast cancer metastasis [63,64]. Moreover, ELMO1 has been identified as a modifier of breast cancer risk for BRCA mutation carriers [65]. Rac1 was shown to be overexpressed in proliferative breast disease, pre-invasive and invasive breast carcinoma, as well as lymph node metastases [66,67]. It was also shown to be implicated in the molecular mechanism of cancer metastasis driven by episodes of hypoxia and re-oxygenation [68], and its overexpression was found to be associated with the aggressive form of breast cancer [67,69]. Rac1 is a member of the Ras superfamily of small guanosine triphosphatases (GTPases) that acts as molecular switches to control cytoskeletal rearrangements and cell growth. Rac1 activity, as a modulator of the cytoskeleton, is critical for a number of normal cellular activities, including phagocytosis, mesenchymal-like migration, axonal growth, adhesion and differentiation of multiple cell types, as well as reactive oxygen species (ROS)-mediated cell killing (reviewed in [70]). Rac1 also plays a major role in the moderation of other signaling pathways involved in cellular growth and cell cycle regulation [71], the formation of cell–cell adhesions [72], and the process of contact inhibition [73]. These Rac1-mediated activities appear central to the processes that underlie malignant transformation, including tumorigenesis, angiogenesis, invasion, and metastasis. Building on the role of the cytoskeleton in cell fusion, a recent study in drosophila showed that cell fusion was driven by the mechanical tension of cell membranes [74]. They demonstrated that, during cell–cell fusion, the receiving fusion partner mounts a non-muscle Myosin II (MyoII)-mediated mechanosensory response to the invasive force from the attacking cell. MyoII is recruited to the fusogenic synapse because of its intrinsic ability to sense mechanical strains in the actin network, whereas chemical signaling from membrane-associated molecules Rho and Rok increased the amount of activated MyoII to amplify the response. The accumulated MyoII generated cortical tension required to resist podosome-like structure invasion, thereby promoting membrane juxtaposition and fusion [74]. The possible mechanistic link between apoptosis and cytoskeletal activation to encourage fusion is delineated in Figure 1. Taken together, one could identify therapeutic targets that may be less promiscuous than directly tapping the cytoskeleton, but still potentially successful in limiting cell–cell fusion in the tumor bed. 

While cell fusion is observed in various pathological conditions, it is also a fundamental requirement in numerous developmental and physiological processes in eukaryotes. These processes include the homotypic fusion of myoblasts, trophoblasts [75], and macrophages [75,76], as well as the heterotypic fusion of gametes [75]. Inhibiting cancer cell fusion could be harmful for these naturally occurring processes, and should be considered while devising treatment to inhibit cancer cell fusion and prevent metastasis. Inhibitors of fusion should be cell-specific to target only the tumor bulk, be delivered and sequestered locally, or used at a dose that is not toxic for the normal biological events.

As we learn more of the processes involved in cell fusion in cancer, better strategies should emerge for targeting critical steps in fusion and hybrid formation for the prevention of metastasis and better cancer management. Given the growing body of observational studies linking spontaneous fusion and cancer progression, there is valid evidence to justify pushing the field forward by way of mechanistic studies. As a result, the effective inhibition of fusion might be formally tested as a means to avoid tumor metastasis and drug resistance.
